# Prospects for *de novo* phasing with *de novo* protein models

**DOI:** 10.1107/S0907444908020039

**Published:** 2009-01-20

**Authors:** Rhiju Das, David Baker

**Affiliations:** a Department of Biochemistry, University of Washington, Seattle, WA 98195, USA

**Keywords:** structure prediction, molecular replacement, *de novo* phasing

## Abstract

The prospect of phasing diffraction data sets ‘*de novo*’ for proteins with previously unseen folds is appealing but largely untested. In a first systematic exploration of phasing with Rosetta *de novo* models, it is shown that all-atom refinement of coarse-grained models significantly improves both the model quality and performance in molecular replacement with the *Phaser* software. 15 new cases of diffraction data sets that are unambiguously phased with* de novo* models are presented. These diffraction data sets represent nine space groups and span a large range of solvent contents (33–79%) and asymmetric unit copy numbers (1–4). No correlation is observed between the ease of phasing and the solvent content or asymmetric unit copy number. Instead, a weak correlation is found with the length of the modeled protein: larger proteins required somewhat less accurate models to give successful molecular replacement. Overall, the results of this survey suggest that *de novo* models can phase diffraction data for approximately one sixth of proteins with sizes of 100 residues or less. However, for many of these cases, ‘*de novo* phasing with *de novo* models’ requires significant investment of computational power, much greater than 10^3^ CPU days per target. Improvements in conformational search methods will be necessary if molecular replacement with *de novo* models is to become a practical tool for targets without homology to previously solved protein structures.

## Abstract 2

In a first systematic exploration of phasing with Rosetta *de novo* models, it is shown that all-atom refinement of coarse-grained models significantly improves both the model quality and performance in molecular replacement with the *Phaser* software.

## 1. Introduction

Molecular replacement has become one of the most widely used tools for solving the crystallographic phase problem for protein diffraction data sets. With widely available software, rapid phasing is possible if models with structural similarity to the crystallized protein are available (Blow & Rossmann, 1961 ▶). As the data bank of solved protein crystal structures continues to expand and as comparative modeling methods become increasingly sophisticated (Schwarzenbacher *et al.*, 2004 ▶; Giorgetti *et al.*, 2005 ▶; Raimondo *et al.*, 2007 ▶; Qian *et al.*, 2007 ▶), the use of molecular replacement is likely to continue to grow.

In recent years, a new frontier for molecular replacement has come into view. A number of diffraction data sets have now been phased ‘*de novo*’, *i.e.* in the absence of evolutionary information from structural homologs or experimental data from methods such as NMR. For a tetrameric coiled coil (Howard *et al.*, 2007 ▶) or a heptameric membrane helix assembly (Strop *et al.*, 2007 ▶), the stereotypical conformation of helices and assumptions regarding the internal symmetry of the complexes have been sufficient to produce successful molecular-replacement templates. [There has also been a long history of phasing nucleic acid crystals with ideal double helices; see, for example, Szep *et al.* (2003) ▶.] For the more general case of an asymmetric protein, our group, in collaboration with the developers of *Phaser* (McCoy, 2007 ▶), has recently presented an example in which the diffraction data for target T0283 in the 2006 Critical Assessment of Structure Prediction were phased by a *de novo* blind model produced with the Rosetta high-resolution prediction methodology (Qian *et al.*, 2007 ▶). These examples suggest that *de novo* modeling is beginning to pass a quite stringent test for accuracy, as was eloquently anticipated by Petsko eight years ago (Petsko, 2000 ▶). Perhaps more importantly, a template-free approach holds practical promise for assisting crystallographic phasing for the substantial number of targets for which structural homologues or other experimental data are not available. However, the number of existing examples remains anecdotal; a much larger set of successful *de novo* phasing solutions is required to delineate the current capabilities and limitations of the method.

In this study, we systematically explore three aspects of this molecular-replacement approach, which we have termed ‘*de novo* phasing with *de novo* models’. We first test whether all-atom refinement of initial low-resolution models is critical for successful molecular replacement, carrying out a benchmark on 30 diffraction data sets. Secondly, we test whether bringing to bear a large amount of computational power (well over 1000 CPU days per target) increases the rate of successful phasing with Rosetta *de novo* models. Finally, we inspect these benchmark results to determine whether particular parameters such as solvent content and the number of copies in the asymmetric unit render diffraction data sets more amenable or more difficult for molecular replacement. With more than a dozen new examples of successful *de novo* phasing, this study presents a first portrait of what can and cannot be achieved when combining state-of-the-art *de novo* structure modeling and state-of-the-art molecular-replacement methods.

## 2. Is all-atom refinement necessary for *de novo* phasing?

The critical advance that has enabled blind *de novo* methods to produce high-resolution predictions (better than 2 Å C^α^ r.m.s.d. from the crystal structure) has been the refinement of initial coarse-grained models in the context of a physically realistic all-atom force field (Rohl *et al.*, 2004 ▶; Kuhlman *et al.*, 2003 ▶; Bradley *et al.*, 2005 ▶; Das *et al.*, 2007 ▶). [We note here that ‘refinement’ refers to the optimization of model conformations in the absence of experimental data and should not be confused with refinement of coordinates based on diffraction data, as occurs during crystallographic structure determination (Murshudov *et al.*, 1997 ▶).] The Rosetta all-atom refinement method is computationally expensive. The sharp penalties associated with atom–atom steric clashes require the extensive minimization of all the protein degrees of freedom after each exploratory perturbation of the side-chain or backbone con­formation. With current processors, the Rosetta algorithm requires on the order of an hour to refine a protein model with a length of 100 residues, nearly twice as long as the initial low-resolution conformational search (Das *et al.*, 2007 ▶).

On one hand, this expensive procedure produces very little change in the structure, with the protein backbone typically shifting by less than 2 Å. On the other hand, the final all-atom score is typically far better at discriminating near-native conformations from non-native models than low-resolution force fields and is thus critical for selecting a small number of blind high-resolution predictions from pools of thousands of low-resolution models. For the CASP7 blind trials in 2006, *de novo* predictions from our group required the use of a distributed network of tens of thousands of volunteer computers, Rosetta@home, to carry out all-atom refinement on the models generated for nearly 100 targets (Das* et al.*, 2007 ▶).

However, for applications to the crystallographic phase problem such refinement may not be necessary. Automated robust molecular-replacement software packages such as *Phaser* permit the screening of thousands of models in a single night on current computer clusters. It may be that models containing only the N, C^α^, C^β^, C and O heavy atoms produced by the first low-resolution conformational search of Rosetta can be selected based on their performance in likelihood-based molecular replacement, without a computationally expensive Rosetta all-atom refinement occurring in between. For example, while the diffraction data for CASP target T0283 could be phased with a high-resolution blind prediction (Qian *et al.*, 2007 ▶), we subsequently discovered that automated and nearly complete rebuilding of the structure could also be achieved by molecular replacement with a coarse-grained low-resolution model, albeit one selected based on knowledge of the crystal structure (unpublished results). Can low-resolution computationally inexpensive models be used in general for molecular replacement?

To investigate this question, we carried out a benchmark of phasing with low-resolution and high-resolution models, focusing on sequences of length ∼100 residues or less from an in-house *de novo* modeling benchmark. For each of these sequences, the structures of targets and of proteins homologous in sequence or structure were removed from the fragment libraries used to generate the Rosetta models (Bradley *et al.*, 2005 ▶), thus mimicking a real-world trial in which templates would not be available for a new protein target. The set of benchmark sequences was pre-filtered based on small-scale low-resolution modeling runs indicating that Rosetta conformational sampling could produce models within 3 Å C^α^ r.m.s.d. from experimental structures, with the hope that aggressive sampling and high-resolution refinement would yield structures within the ∼1.5 Å C^α^ r.m.s.d. accuracy bound that is widely considered to be necessary for accurate molecular replacement (Chen *et al.*, 2000 ▶). (Targets for which Rosetta cannot currently achieve this accuracy were assumed to be beyond the scope of successful molecular replacement and remain challenges for improving Rosetta’s low-resolution conformational sampling.) For 16 of the 32 considered sequences, experimental structure factors were available in the Protein Data Bank for crystals containing the protein sequence of interest and no other macromolecule chains. For several of the sequences, crystals in different space groups were available and the diffraction data with the highest resolution available for each space group were chosen. A total of 30 diffraction data sets were assembled into a final benchmark for *de novo* phasing (Table 1 ▶). For each sequence, we invested 100 CPU days per target for low-resolution modeling and 100 CPU days per target for high-resolution modeling. This computational effort is on a scale that is feasible with computer clusters available at most research institutions and leads to 1 × 10^4^ to 4 × 10^4^ low-resolution models for each sequence. Fewer high-resolution models (3 × 10^3^ to 1 × 10^4^) are obtained with the same computational power owing to the expense of all-atom refinement. The modeling was carried out on Rosetta@home (v.5.96).

Molecular-replacement trials were carried out with *Phaser* 1.3.3, available as part of the *CCP*4 software suite (McCoy, 2007 ▶), using the default *Phaser* parameters and inputting a putative C^α^ r.m.s.d. uncertainty of 1.5 Å for each model. To save on computational expense at this phasing step, we targeted a subset of 200 models filtered with the best energies and as a control a group of 200 randomly chosen models.

The criteria we used to determine whether the molecular-replacement solution was unambiguous and accurate were twofold. Firstly, the *Phaser* translation-function *Z* score (TFZ) of the model was required to be five standard deviations beyond the mean TFZ score seen in the randomly chosen models; a universal absolute cutoff for TFZ did not seem to be appropriate because some diffraction data sets showed uniformly depressed or elevated TFZ values for random models. For the *P*1 space group, the rotation-function *Z* score (RFZ) was monitored instead of TFZ. If more than one molecule was present in the asymmetric unit, iterative and automated *Phaser* searches for all the molecules were carried out using the software’s default settings and the TFZ score for the final model was monitored. Secondly, the rotational orientation of the model needed to be correct; we required that at least half of the C^α^ atoms in the model were positioned within 2 Å of a C^α^ atom in the native structure after translating the centers of mass to the origin and applying the different rotation matrices associated with the crystal’s point group.

The results of this benchmark, given in Table 1 ▶, strongly indicate the importance of all-atom refinement in carrying out molecular replacement with *de novo* models. Out of 30 data sets, low-resolution models passing these criteria for un­ambiguous phasing were found in only one case. The rate of successful molecular replacement was significantly greater among the smaller but more accurate pools of high-resolution all-atom-refined models, with nine cases giving success. In the 21 cases in which phasing was not achieved, was success precluded by limits in the applied conformational search or other properties of the protein sequence or the diffraction data set? To derive a broader understanding of the factors that affect the success of *de novo* phasing, we sought a larger and more diverse set of diffraction data sets phased by *de novo* models, as described in the following.

## 3. Large-scale tests

In *de novo* modeling, increasing the amount of computational power enables larger scale conformational searches and higher resolution models that can be selected based on their all-atom energies (Das *et al.*, 2007 ▶). For the protein sequences tested here, very large collections of all-atom refined models were already available from previous benchmark studies on Rosetta@home. With 10^4^–10^5^ CPU days invested in each target, which is more than one hundred-fold greater computational power than in the tests above, between 1 × 10^5^ and 5 × 10^7^ models could be generated for each target. As above, to save computational time for molecular replacement (which has not been implemented for distributed computing), *Phaser* runs were carried out on 200 models with the lowest all-atom energy and 200 randomly chosen models.

**Figure 1:**
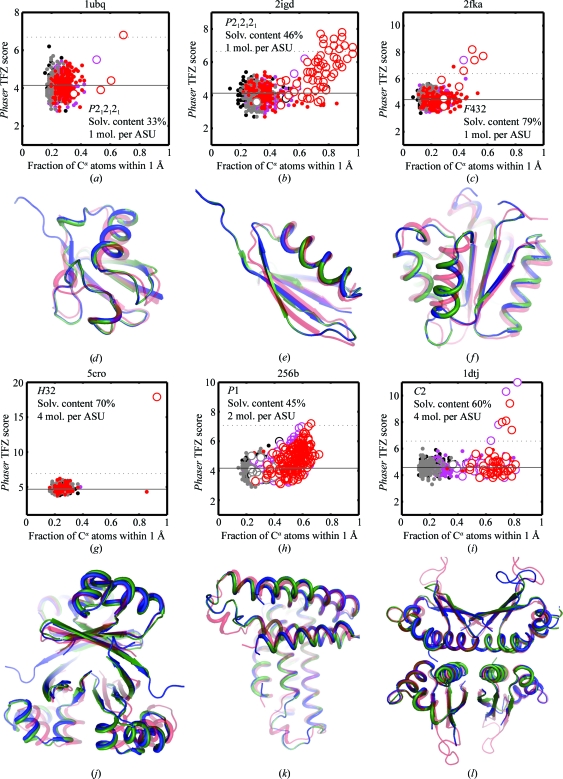
New examples of successful molecular replacement with Rosetta *de novo* models. (*a*)–(*c*) and (*g*)–(*i*) display correlations of *Phaser* translation-function *Z* score (TFZ) with model accuracy (the fraction of C^α^ atoms within 1 Å of the crystal structure). For each target, the displayed subsets are 200 randomly selected all-atom refined models (black) and 200 models with lowest energy from the 100 CPU-day low-resolution set (gray), from the 100 CPU-day all-atom refined set (magenta) and from the large-scale all-atom refined set (red). The solid line and dashed line display the mean TFZ scores and a cutoff value five standard deviations above the mean TFZ, respectively, in the randomly chosen models. Larger open circles indicate *Phaser* solutions with correct orientations in the unit cell (see text). (*d*)–(*f*) and (*j*)–(*l*) give overlays corresponding to each plot in (*a*)–(*c*) and (*g*)–(*i*), respectively, of the least accurate model that passes the TFZ cutoff value (red, partly transparent), nearly complete models built by *ARP*/*wARP* after molecular replacement (green) and the crystal structure (blue). In some cases, the modeled sequence did not include terminal segments present in the crystal structures [see red structures in (*d*)–(*f*) and (*j*)–(*l*)].

With the application of greatly enhanced computational power, the number of diffraction data sets unambiguously phased by *de novo* models increased significantly from nine to 15. Examples of these phasing successes are shown in Fig. 1 ▶. Furthermore, for each of the all-atom refined cases, we put the models with the highest *Phaser* TFZ scores through *flex-wARP* (Cohen *et al.*, 2008 ▶), the latest version of the automated coordinate-building package *ARP*/*wARP*, using default parameters (Perrakis *et al.*, 1999 ▶). This widely used method can produce complete and accurate models if the quality of the beginning molecular-replacement solution was high (Cohen *et al.*, 2008 ▶). In each of the cases investigated, *flex-wARP* was able to successfully and accurately build and sequence-assign the majority of the protein residues (see Fig. 1 ▶). The data sets that were successfully phased span the full spectrum of crystallographic space groups, from the most common, *P*2_1_2_1_2_1_ (Figs. 1 ▶ *a* and 1 ▶ *b*), to rarer groups such as *H*32 (Fig. 1 ▶ *g*) to groups with fewer symmetry operators such as *P*1 (Fig. 1 ▶ *h*). Furthermore, crystals with solvent contents at the lowest end of the probed diffraction data sets (*e.g.* 33%; see Fig. 1 ▶ *a*) were phased. Finally, crystals with multiple copies in the asymmetric unit appeared to pose no fundamental barrier to *Phaser*’s automated multi-copy search (Figs. 1*g*–1*i*); use of a newer version of *Phaser* that takes into account noncrystallographic symmetry, which is a frequent property of protein crystals (Kleywegt & Read, 1997 ▶), may even further enhance the success rate.

Finally, we investigated potential causes for the failure of molecular replacement for the remaining 15 of the 30 diffraction data sets. On one hand, these cases may have been refractory to phasing by *de novo* models owing to artefacts in the Rosetta all-atom force field, *e.g.* its tendency to extend surface side chains into solution or its use of ideal bond lengths and bond angles. On the other hand, *de novo* phasing may have failed simply as a consequence of the unavailability of sufficiently accurate structures in the tested pools of Rosetta models^1^. To test these hypotheses, we carried out phasing runs on a more native-like set of Rosetta all-atom refined models that had been prepared with the *de novo* protocol constrained with information derived from the native structure. Additional constraints were imposed to favor the native assignment of each residue’s backbone torsions in coarse regions of the Ramachandran plot (Blum *et al.*, 2007 ▶; D. Kim & D. Baker, manuscript in preparation). From the approximately 50 000 models in each of these sets, a subset of the 40 lowest C^α^ r.m.s.d. models were subjected to *Phaser* molecular replacement. With this more native-like population of models, 26 of the 30 diffraction data sets could be phased successfully (Table 1 ▶). The four remaining data sets could be phased by models generated by all-atom refinement of the native protein structure after idealization of bond lengths and bond angles. These results suggest that there are no intrinsic artefacts in the current Rosetta all-atom force field that fundamentally confound molecular replacement, but that improved conformational search methods will be required if molecular replacement with *de novo* models is to become a practical routine tool.

Overall, half of the cases tested in this benchmark could be phased *de novo* using a small set of the lowest energy all-atom models. Because approximately one third of proteins in the tested size range appear to be predictable at high resolution (Bradley *et al.*, 2005 ▶; Das *et al.*, 2007 ▶), we estimate that one sixth of diffraction data sets for proteins with new folds and sizes of 100 residues or less can be phased with existing methods. We emphasize, however, that the presented successful molecular-replacement cases have made use of many thousands of CPU days per target made available through distributed computing. Limiting the computational expense from >10 000 CPU days to 100 CPU days significantly reduced the number of phased data sets (from 15 to nine). Omission of the all-atom refinement step led to an even more significant drop (from nine successes to one success). Additional strategies to explore in the future include the use of alternative measures of phasing success beyond the *Phaser* TFZ score, such as the reduction in *R*_free_ upon likelihood-based refinement of the molecular-replacement solution against the diffraction data (Murshudov *et al.*, 1997 ▶), as well as alternative processing of *de novo* models before phasing, such as incorporating estimates of model uncertainty into the *Phaser* likelihood calculation.

## 4. Tentative ‘rules of thumb’ for *de novo* phasing

Based on the phasing results presented so far, neither a low solvent content nor the presence of multiple protein copies in the asymmetric unit appears to be an insurmountable barrier for phasing with *de novo* models. It may be possible, however, that these factors or other properties of the diffraction data set can render the phase problem more difficult or more straight­forward to solve by molecular replacement. With more than 500 Rosetta models tested in *Phaser* molecular replacement for each of 30 diffraction data sets, this study permits an initial exploration of factors that might correlate with the ease with which *de novo* models lead to successful molecular replacement.

We estimated this ease of phasing for each diffraction data set by assessing the minimal model accuracy *F*_1 Å_ required to achieve a significant TFZ score and correct orientation of the model in the unit cell (see above). These estimates (Table 1 ▶) are intrinsically noisy, since for many diffraction data sets only a few models were found to give successful *Phaser* hits. However, these minimal *F*_1 Å_ values provide useful initial estimates to search for the strongest (and thus most practically useful) correlations of crystal parameters with the ease of phasing.

**Figure 2:**
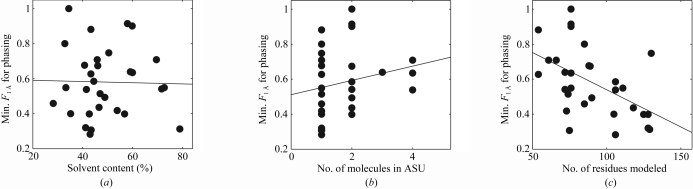
Dependence of *de novo* phasing on crystallographic parameters. The ease of phasing is estimated as the minimal accuracy required for successful molecular replacement (minimum *F*_1 Å_, the fraction of C^α^ atoms within 1 Å of the crystal structure). No correlation is observed with the crystal solvent content (*a*) or the number of molecules in each asymmetric unit (*b*), but a statistically significant correlation is found with the number of residues in the molecular-replacement model (*c*). See also Table 2 ▶.

Table 2 ▶ lists the correlations of these minimal *F*_1 Å_ values with ten parameters associated with the crystallographic data sets, from the number of reflections available to the resolution of the diffraction data to the solvent content of the data. No correlation was detected between higher solvent content and ease of phasing (Fig. 2 ▶ *a*). For example, the benchmark includes four diffraction data sets for ubiquitin, in which the same pool of Rosetta models was tested for phasing, and the data set with the lowest solvent content (PDB code 1ubq) was the only one for which a *de novo* model gave a successful *Phaser* solution. Further, there was no correlation of ease of phasing with the number of molecules in the asymmetric unit (Fig. 2 ▶ *b*); as noted above, use of the next-generation *Phaser* may in fact soon further ease the confident and rapid phasing of crystals with multi-copy asymmetric units.

Of the other parameters tested (Table 2 ▶), only one, the molecular weight of the monomer, gave a statistically significant correlation (*P* < 10^−1^) with minimal *F*_1 Å_ values (Fig. 2 ▶ *c*). Larger macromolecules give crystals that are easier to phase (*P* < 5.7 × 10^−4^). As was pointed out by Randy Read (personal communication) in an informal discussion, more low-resolution data are available for each molecule for constraining its rotation and translation in the unit cell. Indeed, the correlation of more straightforward molecular replacement with larger macromolecules is abundantly illustrated by other articles in this issue, with the successful phasing of the ribosome, the fatty-acid synthase complex (Jenni & Ban, 2009 ▶) and other massive complexes with partly accurate low-resolution models. In our case, as the conformational search in *de novo* modeling becomes (exponentially) more difficult with protein length, it is gratifying that successful molecular replacement may require somewhat less accurate models for longer chains.

## 5. Summary and prospects

Molecular replacement with *de novo* models of protein structures is a potentially useful new tool for phasing diffraction data sets for which experimental phasing has failed and structural homologs cannot be identified from sequence alone. In this study, we have explored the necessity of all-atom refinement of *de novo* models, the general rate of success of this *de novo* phasing method and the properties of a diffraction data set that aid or complicate the method.

Firstly, all-atom refinement of coarse-grained Rosetta models and the application of increasing computational power appear to significantly bolster both model quality and performance in *Phaser* molecular replacement. Secondly, we have presented 15 new cases of diffraction data sets for a wide range of protein folds that have been phased by *de novo* models. These results suggest that approximately one sixth of existing diffraction data sets for small-sized proteins of new folds may be phased with current algorithms if a large amount of computational power is available. Finally, the ease of phasing appears to be poorly correlated with the crystal solvent content or the number of molecules in the asymmetric unit, but is correlated with molecular weight: larger proteins require somewhat less accurate models for successful molecular replacement. As the conformational search for Rosetta modeling improves or is aided by limited additional experimental information (from, for example, NMR chemical shifts; see Cavalli *et al.*, 2007 ▶; Shen *et al.*, 2008 ▶), molecular replacement with *de novo* models can perhaps join molecular replacement with structural homologues as a practical tool for phasing protein diffraction data sets.

The utility of phasing with *de novo* models will be best demonstrated by cases in which blind predictions provide molecular-replacement solutions for diffraction data that have not been phased by other means. We are currently collaborating with structural genomics initiatives and traditional biology laboratoriess in an effort to identify and solve such data sets. In the meanwhile, *a posteriori* crystallographic phasing continues to be a powerful and stringent test of *de novo* modeling algorithms.

## Figures and Tables

**Table 1:** *De novo* phasing benchmark

								Minimum *F*_1 Å_ of an unambiguous *Phaser* solution†				
Structure factors	Model sequence‡	Space group	No. of residues in model	No. of molecules in ASU	Solvent content (%)	No. of models, 100 CPU days§	No. of models, large-scale¶	Low-resolution models, 100 CPU days††	All-atom models, 100 CPU days††	All-atom models, large-scale††	Models, native constraints‡‡	Overall§§
1be7	1bq9	*H*3	51	1	43	3.5 × 10^5^	1.7 × 10^7^	—	—	—	0.882	0.882
1bq9	1bq9	*P*2_1_2_1_2_1_	51	1	43	3.5 × 10^5^	1.7 × 10^7^	—	—	—	0.627	0.627
2igd	1pgx	*P*2_1_2_1_2_1_	55	1	46	2.7 × 10^5^	4.2 × 10^5^	—	—	0.745	0.891	0.709
5cro	5cro	*H*32	55	4	70	2.6 × 10^5^	7.4 × 10^5^	—	—	0.927	0.982	0.709
1hz5	1hz6	*P*3_2_21	61	2	72	2.3 × 10^5^	7.3 × 10^5^	—	0.541	0.656	0.787	0.541
1hz6	1hz6	*P*2_1_2_1_2_1_	61	3	59	2.3 × 10^5^	7.3 × 10^5^	—	0.672	0.689	0.836	0.639
1a32	1a32	*P*2_1_2_1_2_1_	65	1	41	2.8 × 10^5^	2.8 × 10^5^	—	0.754	0.708	0.800	0.677
1ctf	1ctf	*P*4_3_2_1_2	68	1	47	2.4 × 10^5^	3.2 × 10^5^	—	—	—	0.882	0.515
1aar	1ubi	*P*1	71	2	35	2.0 × 10^5^	5.4 × 10^7^	—	—	—	—	1.000
1f9j	1ubi	*I*4_1_22	71	2	60	2.0 × 10^5^	5.4 × 10^7^	—	—	—	—	0.901
1ubq	1ubi	*P*2_1_2_1_2_1_	71	1	33	2.0 × 10^5^	5.4 × 10^7^	—	—	0.690	0.662	0.549
2fcq	1ubi	*P*4_3_32	71	2	58	2.0 × 10^5^	5.4 × 10^7^	—	—	—	—	0.915
2ojr	1ubi	*P*3_2_21	71	1	73	2.0 × 10^5^	5.4 × 10^7^	—	—	—	0.549	0.549
1dt4	1dtj	*P*4_2_2_1_2	74	1	54	2.8 × 10^5^	4.9 × 10^5^	0.649	0.622	0.500	0.635	0.419
1dtj	1dtj	*C*2	74	4	60	2.8 × 10^5^	4.9 × 10^5^	—	0.635	0.716	0.811	0.635
1ig5	1ig5	*P*4_3_2_1_2	75	1	43	2.3 × 10^5^	8.3 × 10^6^	—	—	—	0.307	0.307
1cm3	1opd	*P*2_1_	85	1	28	2.3 × 10^5^	8.4 × 10^6^	—	—	—	0.753	0.459
1opd	1opd	*P*1	85	1	33	2.3 × 10^5^	8.4 × 10^6^	—	—	—	0.800	0.800
1a19	1a19	*I*4_1_	89	2	49	1.7 × 10^5^	7.0 × 10^6^	—	—	—	0.494	0.494
2hxx	1a19	*C*2	89	2	46	1.7 × 10^5^	7.0 × 10^6^	—	—	—	0.674	0.674
1mb1	1bm8	*P*4_1_2_1_2	99	1	51	1.6 × 10^5^	9.2 × 10^5^	—	—	—	—	0.747
2hsh	1aiu	*C*2	105	1	35	1.5 × 10^5^	4.4 × 10^5^	—	—	0.400	0.600	0.400
1m6t	256b	*C*222_1_	106	1	43	1.8 × 10^5^	1.5 × 10^5^	—	0.453	0.443	0.491	0.283
256b	256b	*P*1	106	2	45	1.8 × 10^5^	1.5 × 10^5^	—	—	0.660	0.594	0.585
2bc5	256b	*P*2_1_2_1_2_1_	106	4	42	1.8 × 10^5^	1.5 × 10^5^	—	0.538	—	0.689	0.538
1elw	1elw	*P*4_1_	117	2	47	1.5 × 10^5^	1.1 × 10^5^	—	0.453	0.521	0.897	0.436
1ab6	2chf	*P*3_1_	128	2	57	1.2 × 10^5^	3.5 × 10^6^	—	—	0.508	0.398	0.398
2fka	2chf	*F*432	128	1	79	1.2 × 10^5^	3.5 × 10^6^	—	0.430	0.359	0.367	0.313
3chy	2chf	*P*2_1_2_1_2_1_	128	1	41	1.2 × 10^5^	3.5 × 10^6^	—	—	—	0.492	0.320
6chy	2chf	*P*2_1_2_1_2_1_	128	2	43	1.2 × 10^5^	3.5 × 10^6^	—	—	0.398	0.422	0.398

† *F*_1 Å_ is a measure of model accuracy: the fraction of C^α^ atoms within 1 Å of the crystal structure of the modeled sequence. A dash (—) indicates that no models were found within the specified subset that gave an unambiguous *Phaser* solution.

‡ The Rosetta-modeled sequences were taken from an in-house curated benchmark used to test *de novo* modeling; in some cases the sequence does not include terminal segments (typically loops) or particular mutations present in the crystallized sequence.

§ Results of 100 CPU days per target without all-atom refinement, as is typically achievable by a state-of-the-art computer cluster; application of the same computational effort but including all-atom refinement led to pools of approximately one third the size.

¶ Results from 10^4^–10^5^ CPU days per target, with all-atom refinement, as is achievable with distributed computing.

†† Out of each pool of *de novo* models, the 200 models with best energies were tested for molecular replacement.

‡‡ Out of pools of approximately 50 000 models produced with the *de novo* method constrained with coarse native information for the backbone torsion angles, 40 models with the lowest C^α^ r.m.s.d. were tested for molecular replacement.

§§ Minimum *F*_1 Å_ that led to an unambiguous *Phaser* solution among all models tested in this study, including an additional 50 models with the lowest C^α^ r.m.s.d. to the crystal structure for each set (results not separately shown). These values are used as estimates of the ‘ease of phasing’ for each data set (see Table 2 ▶).

**Table 2:** Correlation of different crystallographic parameters with the minimal accuracy of a *de novo* model required to phase the 30 diffraction data sets in Table 1 ▶

Crystallographic parameter	*r*^2^	*P* value†
No. of modeled residues	−0.592	5.7 × 10^−4^
Highest resolution reflection	0.232	0.22
Lowest resolution reflection	−0.229	0.22
No. of copies in asymmetric unit	0.200	0.29
No. of reflections	−0.146	0.44
Matthews coefficient (*V*_M_)	−0.095	0.62
No. of reflections > 4 Å	−0.091	0.64
No. of reflections > 6 Å	−0.079	0.68
No. of residues in asymmetric unit	−0.038	0.84
Solvent content	−0.022	0.91

† Correlations are to *F*_1 Å_, the fraction of C^α^ atoms within 1 Å of the crystal structure, of the least accurate model that gives an unambiguous *Phaser* hit (see Table 1 ▶).

## Footnotes

1 In a further test, we subjected the 50 low-resolution models with lowest C^α^ r.m.s.d. to the crystal structure from each pool to molecular replacement in order to estimate an upper bound on the number of successes that might be possible if all the available models could be tested. For the low-resolution set, this search increased the number of data sets with unambiguous *Phaser* solutions from one to four. However, in both the 100 CPU day and large-scale all-atom model sets, the number of successes increased by only one (from nine to ten and from 15 to 16, respectively). These results indicate that for most applications molecular-replacement trials need only to be carried out on a limited set of the best energy models if the energies are assessed by Rosetta all-atom refinement.
